# Effect of Soybean Soluble Polysaccharide on the Formation of Glucono-δ-Lactone-Induced Soybean Protein Isolate Gel

**DOI:** 10.3390/polym11121997

**Published:** 2019-12-03

**Authors:** Qiuyu Lan, Lin Li, Hongmin Dong, Dingtao Wu, Hong Chen, Derong Lin, Wen Qin, Wenyu Yang, Thava Vasanthan, Qing Zhang

**Affiliations:** 1 College of Food Science, Sichuan Agricultural University, Ya’an 625014, China; lanqiuyu211@163.com (Q.L.); lilin2520@163.com (L.L.); DT_Wu@sicau.edu.cn (D.W.); chenhong945@sicau.edu.cn (H.C.); linderong123@aliyun.com (D.L.); qinwen@sicau.edu.cn (W.Q.); 2 Department of Agricultural, Food and Nutritional Science, University of Alberta, Edmonton, AB T6G 2P5, Canada; hongmin1@ualberta.ca (H.D.); tv3@ualberta.ca (T.V.); 3 Key Laboratory of Crop Ecophysiology and Farming System in Southwest, Ministry of Agriculture/Sichuan Engineering Research Center for Crop Strip Intercropping System, Chengdu 611130, China; mssiyangwy@sicau.edu.cn

**Keywords:** Soybean protein isolate, Soybean soluble polysaccharide, Gel, Glycosylation, Microstructure

## Abstract

The effect of soybean soluble polysaccharide (SSPS) on the formation of glucono-δ-lactone (GDL)-induced soybean protein isolate (SPI) gel was investigated. Electrophoretic analysis showed the SSPS did not change the electrophoretic behavior of SPI during the formation of SPI gel. However, infrared analysis indicated the β-sheet content increased, and the contents of random coil and α-helix decreased in both cooked SPI and SPI gel. The SSPS and SPI might conjugate via the Maillard reaction according to the results of grafting degree, color change, and infrared analyses. The main interactions during the formation of SPI gel changed from non-covalent to electrostatic interaction after adding SSPS. Sulfhydryl group content also increased in both cooked SPI and SPI gel. The water-holding capacity and gel strength of SPI gel decreased as the SSPS concentration increased. Larger aggregate holes were observed in the microstructure of SPI gel at higher SSPS concentration. Thus, SSPS could covalently conjugate with SPI and influence the formation of hydrogen bonds, disulfide bonds, and electrostatic interaction among SPI molecules to eventually form a loose gel network.

## 1. Introduction

Soy protein isolate (SPI), widely known for its diverse functionalities and health benefits, is commonly utilized in the food industry. The gelation is one of the most important functional properties of soybean protein in food [1]. Generally, after thermal denaturation, unfolding, and molecular aggregation, soybean proteins were cross-linked to form a three-dimensional network gel [2]. It has been reported that many factors can affect the formation of protein gels, such as pH, coagulant, heating method, protein concentration, and addition of sugars [3,4,5]. Therefore, it is valuable to study the effects of other ingredients and treatment conditions on the production of protein-based products.

**Figure 1:**
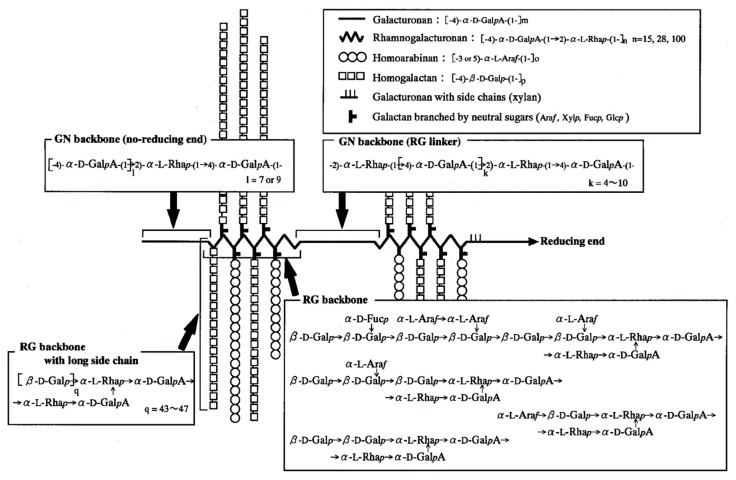
Structural Model of the soybean soluble polysaccharide (SSPS) Molecule (RG, rhamnogalacturonan; GN, homogalacturonan) [6].

Soybean soluble polysaccharide (SSPS), a type of acidic polysaccharide that can be extracted from soy residue (okara), is composed of a main backbone involving long rhamnogalacturonan, branched by β-1,4-galactan, α-1,3 or 1,5-arabinan chains, and short homogalacturonan (Figure 1) [6]. When an aqueous system containing protein and polysaccharide is thermally treated and then cooled, phase separation occurs due to the thermodynamic incompatibility between the two biomacromolecules, forming differentiated structures [7]. As a result, phase separation and aggregation caused by thermal denaturation of proteins occurs during gelation by mixing proteins and polysaccharides. Specifically, during the gelation of protein aqueous solutions containing polysaccharides, interactions between proteins and polysaccharides, such as electrostatic attraction, disulfide bond, hydrogen bond, and hydrophobic interaction between peptide chains and reducing polysaccharides, play an important role in the emerging of physicochemical properties of protein gelation. Furthermore, SSPS can affect the protein aggregation by steric effects because of the highly-branched chain of SSPS and aggregative interactions between the positively charged proteins (pH < pI) and negatively charged polysaccharide at the final pH, which might lead to the degradation of the characteristics of tofu gelation [7,8].

Recently, the influence of polysaccharide on the properties of soy protein isolate (SPI) gels have attracted extensive attention. Gu et al. [9] comparatively investigated the influence of sugars (glucose, lactose, and sucrose) on the characteristics of glucono-δ-lactone (GDL)-induced SPI gels. Results demonstrated that different sugars had different effects on gel strength, water-holding capacity (WHC), and rheological properties of the SPI gels. However, the sugar-added SPI gels exhibited reduced dispersibility, hydrophobicity, and WHC compared to the control. However, Zhao et al. [10] demonstrated the addition of sucrose, raffinose, stachyose, and SSPS improved the hardness, WHC, and rigidity of CaSO_4_-induced soybean protein gels. Previous studies have reported the effect of sugars on the rheological and textural properties of SPI gels. Few studies have been devoted to the formation of acid-induced SSPS-SPI tofu-type gels. The purpose of this study is to provide insights into the effect of SSPS on the formation of GDL-induced SPI gels at a molecular level by correlating with the molecular interactions and the textural and microstructural properties.

## 2. Materials and Methods

### 2.1. Materials and Reagents

SPI was purchased from Shansong Biological Products Co., Ltd., Shandong, China. SSPS was provided by BoMei Biotechnology Co., Ltd. (Hefei, China). GDL was obtained from Xingzhou Medicine Food Co., Ltd. (Anhui, China). O-phthaldialdehyde (OPA), glycine, sodium dodecyl sulfate (SDS), Coomassie Blue G-250, and 8-anilino-1-naphthalene sulfonic acid (ANS) were purchased from Aladdin Co., Ltd. (Shanghai, China). All chemical reagents used in this study were of reagent grade.

### 2.2. Preparation of SPI Gels

Solutions with 10% SPI concentration (*w*/*v*) and different SSPS concentrations (0%, 4%, 8%, 12%, 16%, *w*/*w*) were selected to prepare tofu-type gels. Briefly, mixed powders and deionized water were further mixed using a magnetic blender (G-560E, Scientific Industries, Inc., Bohemia, NY, USA) for 2 min. The suspension was heated at 75 °C for 10 min and was then heated at 90 °C for 10 min. The hot slurry was placed in an ice-water bath until the temperature decreased to 20 °C. GDL (0.30%, *w*/*w*) was dissolved in deionized water (10 mL) and then added as a coagulant into the cooked SPI suspension and stirred for 2 min using a magnetic stirrer [11]. The mixtures (70 g) were divided into cups (100 mL) with a plastic spoon and then incubated at 85 °C for 1 h. The SPI gels were stored at 4 °C in a refrigerator for 12 h before analysis. The cooked SPI suspension, dialyzed SPI suspension, and SPI gels were freeze-dried for the measurements of protein solubility, intrinsic fluorescence, surface hydrophobicity, particle size distribution, protein composition, sulfhydryl group content, degree of grafting, and Fourier transform infrared (FTIR) spectrum.

### 2.3. Electrophoretic Analysis

Sodium dodecyl sulfate-polyacrylamide gel electrophoresis (SDS–PAGE) was performed according to the Laemmli method [12,13] involving 10% separating gel and 4% stacking gel. Lyophilized SPI samples (2 mg/mL) were incubated in SDS–PAGE loading buffer for 1 h, heated at 95 °C for 5 min in a water bath, and then centrifuged at 5000× *g* for 10 min using a TGL-16G 144 centrifuge (Anting Scientific Instrument Co. Ltd., Shanghai, China). Aliquots (10 μL) of the prepared samples were added into the gels. The electrophoresis was run at a constant voltage of 120 V. After separation, the gels were stained by using Coomassie Blue G-250 The gel image was scanned using an image scanner (Smart Gel 130, Saizhi Scientific Co., Beijing, China) and analyzed using Quantity One software (version 4.6.2, Bio-Rad Laboratories, Inc., Hercules, CA, USA).

### 2.4. Measurement of Sulfhydryl Groups

Ellman’s reagent (DTNB) was used to determine the content of free sulfhydryl groups of lyophilized samples of cooked SPI suspension and SPI gels, according to a previous method [14]. Sample solution (2 mg/mL) was mixed with buffer B (4 mmol/L Na_2_EDTA, 0.09 mol/L glycine, and 0.086 mol/L Tris, pH 8.0) and stirred at ambient temperature for 2 h. SPI solutions were centrifuged at 12,000× *g* at 4 °C for 30 min. The supernatant was used for the measurement of the content of sulfhydryl groups. Three milliliters of supernatant was mixed with 0.03 mL Ellman’s reagent solution (4 mg DTNB/mL buffer) and was kept at ambient temperature for 15 min. The absorbance was then measured at 412 nm. The buffer was used instead of sample solutions as a reagent blank. A protein blank was used in which 0.03 mL buffer was replaced by Ellman’s reagent solution.

### 2.5. Determination of Degree of Grafting (DG) of SPI Gels

The DGs of SPI gels were determined indirectly by the OPA method [15]. Samples were diluted in deionized water with a concentration of 3% (*w*/*v*). A 200 μL quantity of sample solution was mixed with 4 mL OPA reagent and was then was maintained at 35 °C for 2 min in a water bath. Absorbance was measured at 340 nm with a Thermo Scientific Microplate Reader (Thermo Scientific, Waltham, MA, USA). SPI without adding SSPS was used as a control. *DG* was calculated according to the following equation:

(1) $$DG(\%)=\frac{A_{0}-A_{1}}{A_{0}}\times 100\%$$

where *A*_0_ is the absorbance of a sample without SSPS, and *A*_1_ is the absorbance of the sample with SSPS addition. All samples were measured in triplicate.

### 2.6. FTIR Spectroscopy Analysis

Based on a previously reported method [16], 2 mg of sample and 200 mg potassium bromide were mixed, ground, and then pressed into a thin disk with a YP-2 tablet press. FTIR spectra of the disks were recorded in a range of 4000–400 cm^−1^ with an FTIR spectrophotometer (Thermo Scientific, Waltham, MA, USA). Each sample was scanned 32 times with a resolution of 4 cm^−1^.

### 2.7. Surface Hydrophobicity Measurement

Surface hydrophobicity of samples was determined according to a previous method [17], using 1-anilino-8-naphthalene-sulfonate (ANS) as a fluorescence probe. The lyophilized samples were dissolved in 0.01 M phosphate buffer (pH 7.0) with a concentration of 2 mg/mL and stirred at room temperature for 2 h. Sixty microliter 8.0 mmol/L ANS was then added to a 3 mL diluted sample solution. A spectrofluorometer (Thermo Scientific, Waltham, MA, USA) was used to measure the relative fluorescence intensity at wavelengths of 365 nm (excitation) and from 400 to 600 nm (emission). Surface hydrophobicity was defined as the maximum fluorescence intensity.

### 2.8. Determination of Protein Solubility

The investigation of noncovalent and covalent interactions involved in the cooking of SPI suspension and the formation of SPI gels was performed by dissolving the samples in different solvents [18]. Solvent systems used to dissolve lyophilized samples with a concentration of 2 mg/mL were as follows: S1, deionized water at pH 8.0 (adjusted with NaOH); S2, Tris–Glycine buffer (0.086 mol/L Tris, 0.09 mol/L glycine, and 4 mmol/L Na_2_EDTA, pH 8.0); and S3, S2 containing 0.5% SDS and 6 mol/L urea. The resultant solutions were incubated at 25 °C for 24 h in a shaking water bath and then were centrifuged at 12,000× *g* and 25 °C for 30 min. The protein concentration of the supernatant was estimated by the Lowry method using a bull serum albumin standard. Absorbance at 650 nm was measured using a Varioskan Flash (Thermo Scientific, Waltham, MA, USA). All determinations were conducted in duplicate. Protein solubility (%) was expressed as 100 times the protein content of the supernatant divided by the total protein content.

### 2.9. Measurement of WHC

Samples with different treatments were subjected to WHC measurement according to a previous method [19] with slight modification. Gel samples (3 g per tube) were measured into 5 mL centrifuge tubes and were centrifuged at 8000 rpm and 25 °C for 20 min in a centrifuge (Jinan Rise Science Technology Co. Ltd., Jinan, China). Tubes were inverted to drain and remove the residual water carefully using dry filter papers after centrifugation. The tubes with gel samples before and after centrifugation were accurately weighted, and WHC was calculated according to the following equation:

(2) $$WHC(\%)=\frac{W_{t}-W_{r}}{W_{t}}\times 100\%$$

where *W*_t_ is the total weight of native SPI gels and *W*_r_ is the weight of SPI gels that were centrifuged.

### 2.10. Analysis of Microstructure

The morphology of SPI gels was observed with a scanning electronic microscope (SEM) (ZEISS EVNO18, Oberkochen, Germany). Before observation, samples were dried to a critical point by a method of Ramlan et al. [20]. Samples were mounted on an aluminum sample plate with two sided carbon tabs and then the samples were coated with a thin layer of gold. The acceleration voltage was set as 20.0 kV, and SmartSEM software (V05.04, Carl Zeiss SMT Ltd., Oberkochen, Germany)) was used to collect the images.

### 2.11. Gel Strength Analysis

The gel strength of SPI gels was measured using GMIA (2013) Standard [21]. SPI gels samples were formed in glass bottles (40 mm diameter 35 mm height; 10 g gel per container), according to the method described in Section 2.2. Gel strength was measured using a TA.XTplus texture analyzer (Stable Micro System, Surrey, UK), with a P/0.5 probe to a target distance (4 mm). The pretest speed, test speed, and post-test speed were 1.0 mm/s. Gel strength was defined as the maximum force used in penetration. Each sample was measured in eight replicates and the average of these readings was recorded as the final value of gel strength.

### 2.12. Color Analysis

The color of SPI gels was determined using a colorimeter (NR 10QC, CIELab, Shenzhen, China). An inbuilt standard white plate was used for the instrument calibration. The measurements were recorded as lightness (L*), redness/greenness (a*), and yellowness/blueness (b*) color scale.

### 2.13. Statistical Analysis

All experiments were made in at least triplicate unless otherwise stated. Data were processed and plotted using Origin Pro 9.1 (Origin-Lab, Northampton, MA, USA). Values given in the tables and figures are the means of triplicates, and error bars indicate the standard deviation. Statistical significance of differences among means was analyzed by analysis of variance test and Duncan’s multiple range tests by SSPS Statistics, version 17.0 (IBM®, Chicago, IL, USA).

## 3. Results and Discussion

### 3.1. Effect of SSPS on the Structure of SPI

#### 3.1.1. Electrophoretic Analysis

**Figure 2:**
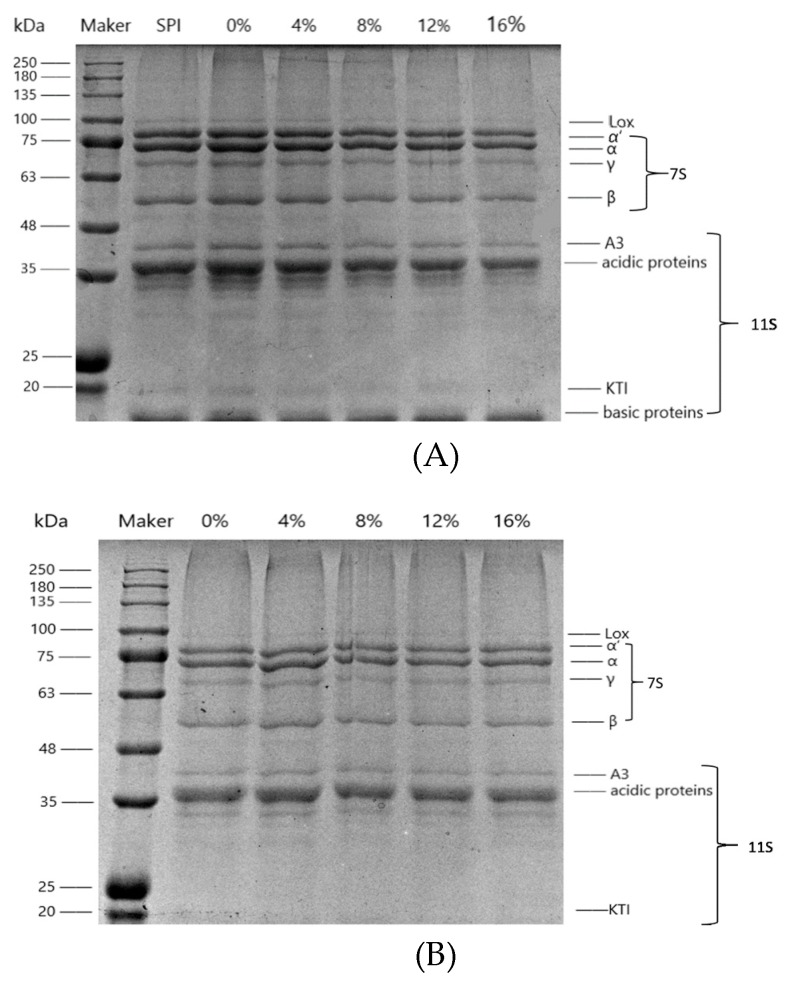
SDS–PAGE patterns of cooked SPI suspension (**A**) and SPI gels (**B**) as the SSPS concentration increased (SDS–PAGE, sodium dodecyl sulfate–polyacrylamide gel electrophoresis; SPI, soybean protein isolate; SSPS, soybean soluble polysaccharide).

The protein composition of cooked SPI suspension and SPI gels at various SSPS concentrations was analyzed by SDS–PAGE. As shown in Figure 2, SSPS in different concentrations did not change the electrophoretic behavior of SPI, indicating that there was no alteration in the primary structure of SPI. Furthermore, it was observed that all of the bands became weak after gelation due to the decline of the solubility of gels in the buffer [21].

#### 3.1.2. Change in Secondary Structure

To analyze the effect of SSPS on the secondary structure of SPI, FTIR spectra were processed by splitting and fitting using PeakFit 4.12 (Systat Software Inc., CA, US). As previously reported, the shape of the amide I band located at 1600–1700 cm^−1^ was used to determine the protein secondary structure [22,23]. As shown in Supplementary Materials Figure S1, all cooked SPI suspension and SPI gel samples showed a similar FTIR spectrum pattern, which meant that there were no new covalent bonds generated. The appearance of a peak at 1100 cm^−1^ was caused by the stretching vibration of O–H and C–C bonds of sugar, indicating that SPI underwent glycosylation modification [24]. Within the range of 3500–3000 cm^−1^, the O–H stretching vibration of cooked SPI suspension and SPI gels caused the emerging of absorption peaks [25]. The O–H stretching vibration of gel samples increased obviously when SSPS concentrations were 12% and 16%, demonstrating that the hydroxyl groups in SSPS were linked to the SPI molecule by a covalent bond to increase the hydroxyl number of SPI.

As exhibited in Table 1, there was a significant increase in the content of the β-sheet and a decrease in the content of α-helix and random coil of cooked SPI suspension and SPI gels when SSPS was added. Generally, the β-sheet is usually buried in the interior of protein [26]. Under acidic conditions, SSPS is shown to prevent the destabilization of SPI [27]. The addition of SSPS led to an increase in the content of the β-sheet of cooked SPI suspension from 22.99% to 42.14%, which might have been due to SSPS preventing the unfolding of the peptide chain of SPI. The decrease in the content of α-helix indicated the breakdown of intermolecular and intramolecular hydrogen bonds [28]. SPI gels tended to form less α-helix with the addition of SSPS, which might have resulted in a loose gel network structure. In addition, the increase in the content of the β-sheet of SPI gels indicated that the gel network structure existed in the form of the β-sheet after the addition of SSPS.

Above all, SSPS did not change the primary structure of SPI but affected the profiles of the secondary structure. The addition of SSPS resulted in increasing the content of the β-sheet and reducing the contents of α-helix and the random coil of cooked SPI suspension and SPI gels, which might have finally resulted in a loose gel network.

### 3.2. Interactions between SSPS and SPI

#### 3.2.1. Change in Protein Solubility

Solubility of protein is the specific behavior of its hydration, which can be improved by increasing the exposed surface charge of polar groups in the depolymerization/subunit extension of protein molecules or molecular polymers [29]. It was reported that urea can break non-covalent bonds, such as hydrogen bonds and hydrophobic interactions, as well as interfere with intermolecular hydrophobic interactions [14]. SDS is known to disrupt hydrophobic protein–protein interactions. Therefore, the solubility of protein in the buffer containing the above reagents can provide relative information for the interaction between the protein molecules. Generally, buffer S2 can interrupt electrostatic interaction, while buffer S3 can interrupt non-covalent bonds (such as hydrogen bond and hydrophobic interaction) [30]. S2–S1 shows the strength of electrostatic interaction, and S3–S2 shows the strength of non-covalent bonds.

According to Table 2, protein solubility of cooked SPI suspension in buffer S2 (Tris-Glycine buffer) was significantly higher than that of cooked SPI suspension in S1 (deionized water at pH 8.0). The different solubility between samples dissolved in S1 and S2 had a significant increase, which was related to the interruption of electrostatic interaction induced by buffer S2. Therefore, it could be assumed that the addition of SSPS resulted in an increase in the electrostatic effect of the heated SPI. Tran et al. [27] examined the surface charge and found SPI and SSPS had alike negative charges at pH 6–8. Protein solubility of cooked SPI suspension first decreased and then increased in S1 and S2 as the SSPS concentration increased, which might be due to the phase separation between SSPS and SPI when the concentration of SSPS was high [27]. Besides, S2–S1 decreased with the increase in the concentration of SSPS, while S3–S2 was higher than the control group, indicating that the addition of SSPS weakened the electrostatic interaction between solutes in the heated solution and enhanced the non-covalent interaction, which was inconsistent with a previous study [27], possibly due to the difference caused by protein denaturation after heating. Gu et al. [9] found that the surface hydrophobicity of heat-treated SPI–sugar dispersions decreased with the increase of sugar at pH 6.8.

In addition, protein solubility of SPI gels in S2 was higher than that of SPI gels in S1, suggesting that the electrostatic interaction was significant for the SPI gel network in all samples. The solubility of the protein in S2 buffer increased obviously after adding SSPS, which meant that electrostatic interaction was the important factor for the formation of the SPI gel network. After the addition of SSPS, S2–S1 was more than 50%, and S3–S2 decreased significantly, indicating that electrostatic interaction was an important factor in the formation of the SPI gel network. In addition, S3–S2 of the SPI gels decreased after the addition of SSPS, reflecting the decrease of the non-covalent interaction in SSPS–SPI gels. This might be due to their different microstructure [11].

#### 3.2.2. Change in Surface Hydrophobicity

Changes in the secondary and tertiary structures of protein affect its spatial conformation and surface charge density. The level of surface hydrophobicity indicates the unfolding degree of protein, which leads to the exposure of non-polar amino acids within the molecule [31]. During the gelation process of polysaccharides and proteins, protein-polysaccharide interactions play an important role, such as the formation of disulfide bonds, hydrogen bonds, and hydrophobic interactions.

**Figure 3:**
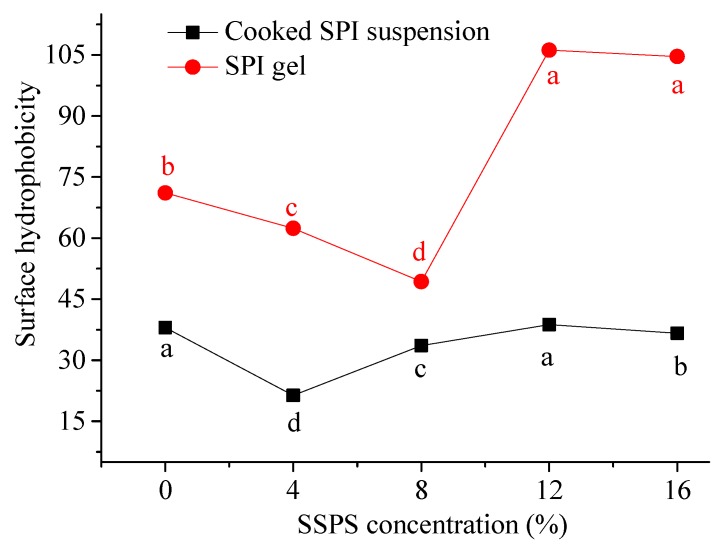
Changes in surface hydrophobicity of cooked SPI suspension and SPI gels (SPI, soybean protein isolate; SSPS, soybean soluble polysaccharide Different lowercase or uppercase meant the significant difference among the different SSPS concentrations, *p* < 0.05).

The surface hydrophobicity of the cooked SPI and SPI gels had a similar change trend (Figure 3), indicating the effect of SSPS on SPI hydrophobic effect showed no remarkable change after the addition of GDL. In addition, the surface hydrophobicity of gels was higher than the cooked SPI suspension. The reason for the increase was the hydrophobic interaction of the neutralized protein molecules became more predominant and induced aggregation after the addition of GDL [29]. It is worth noting that the surface hydrophobicity of cooked SPI and SPI gels fluctuated when the SSPS concentration increased from 0% to 16%. The decrease of surface hydrophobicity of SPI gels may be ascribed to the formation of protein gels through hydrophobic interactions [32]. However, the reason for the increase needs to be further investigated.

#### 3.2.3. Change in Sulfhydryl Group

**Figure 4:**
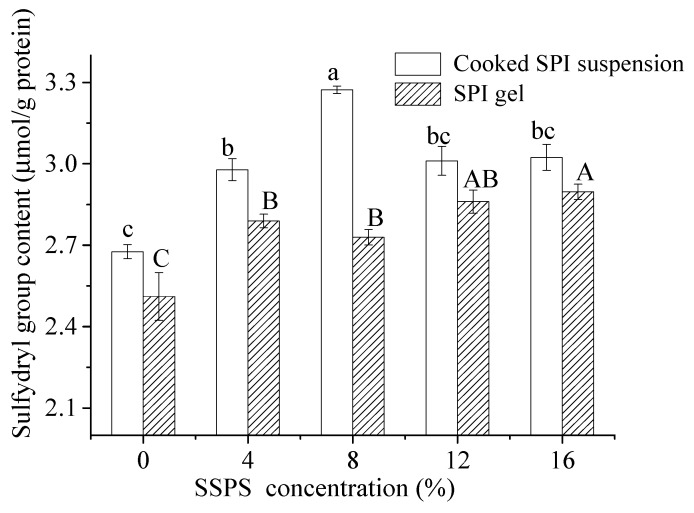
Sulfhydryl group content of the cooked SPI suspension and SPI gels (SPI, soybean protein isolate; SSPS, soybean soluble polysaccharide; a, b, bc, and c refer to the significant difference among the different SSPS concentrations of cooked SPI suspension, *p* < 0.05; A, B, AB, and C refer to the significant difference among the different SSPS concentrations of SPI gels, *p* < 0.05).

The sulfhydryl group is derived from disulfide bonds, which plays an important role in stabilizing the tertiary structure of proteins. Therefore, sulfhydryl group analysis is an indispensable method to explore the structural and functional changes of protein after denaturation. The sulfhydryl group contents of cooked SPI suspension and SPI gels at different SSPS concentrations are shown in Figure 4. The sulfhydryl group contents of cooked SPI suspension samples were higher than those in SPI gel samples. This might be attributed to the gelling after adding GDL where it was buried in the aggregation [33]. As previously described, non-covalent bonds were mainly responsible for maintaining the structure of the GDL-induced gels and few S=S bonds were formed under acidic conditions [18]. The sulfhydryl group contents of SSPS-added gels were higher than those in the blank sample. This suggested that electrostatic interaction between SPI and SSPS in buffer solution might have inhibited the aggregation of stretched peptide chains of SPI or inhibited the formation of disulfide bonds during heating and gelling. Several researchers [9,34] have investigated the fewer number of disulfide bonds associated with lower gel strength.

### 3.3. The Occurrence of the Maillard Reaction between SSPS and SPI

#### 3.3.1. Change in Free Amino Groups

The determination of free amino groups is used to estimate the progress of the Maillard reaction [25,35]. The level of free amino groups determined for SPI gels is exhibited in Table 3. The decreased content of free amino groups could be caused by the glycation reaction between free amino groups of proteins and the reducing end carbonyl group of sugars [36]. As listed in Table 3, the DG of SPI gels increased with the increase of SSPS. Therefore, glycosylation might occur after adding SSPS. Gu et al. [9] have shown that the heating of SPI with glucose and lactose resulted in glycosylation to some extent.

#### 3.3.2. Color Variation

Color is not only an important characteristic of gel quality but also an index to estimate the degree of the Maillard reaction [35]. It is well known that polysaccharides and proteins can form colored compounds in advanced stages of the Maillard reaction; thus, the brown color is a clear indication of the performance of the Maillard reaction. The color of SPI gels is shown in Table 3. As listed in Table 3, the red (a*) and yellow (b*) tones of SPI gels increased as the SSPS concentration increased. Li et al. [35] also reported that a* and b* parameters were found in an increasing trend with increased reaction time. The results further confirmed that SSPS and SPI are linked together via the Maillard reaction.

### 3.4. SPI Gel Properties

#### 3.4.1. WHC

**Figure 5:**
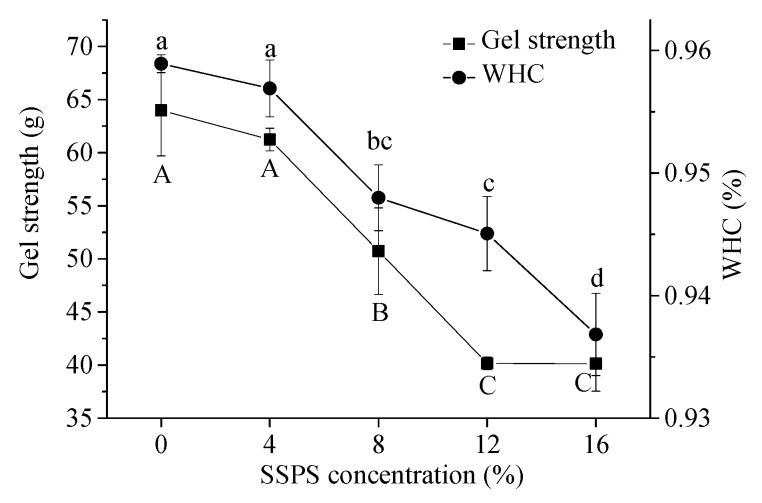
WHC and gel strength of SSPS–SPI gels (C) as the SSPS concentration increased (SPI, soybean protein isolate; SSPS, soybean soluble polysaccharide; WHC, water holding capacity; a, bc, c, and d meant the significant difference among the WHC of SSPS-SPI gels, *p* < 0.05A, B, and C meant the significant difference among the WHC of SSPS-SPI gels, *p* < 0.05).

WHC is an important characteristic of food products, especially for gels [37]. WHC reflects the ability of a gel to effectively immobilize water within its matrices through capillary force [38], which is associated with gel strength and structure. Generally, enhancement in gel strength and homogeneity can improve the WHC. As shown in Figure 5, it was clear that the WHC of SPI gels decreased from 96% to 94% as the SSPS concentration increased. Gu et al. [9] attributed the decreased WHC of SPI–sugar dispersions to the protein–protein and protein–water interactions. Combined with the abovementioned results, it can be concluded that the addition of SSPS leads to the reduction of hydrogen bonds, disulfide bonds, and result in SPI gels bound by the electrostatic force, which forms a loose gel network structure of proteins and finally causes the lower WHC of SPI gels.

#### 3.4.2. Gel Strength

The gel strength is one of the most important characteristics of gels. As exhibited in Figure 4, gel strength of SPI gels decreased from 63.98 to 40.13 g as the SSPS concentration increased. This might be due to SSPS interference in the formation of soybean protein gels. The size of the soluble polysaccharide aggregation phase increased due to the microphase separation. The results were consistent with those findings of solubility that exhibited the addition of SSPS to reduce the neutralization of GDL, and the SEM analysis that was introduced in the following section. First, sugars can prevent proteins from thermal denaturation to affect the gel strength by increasing the temperature of thermal denaturation and changing the bond formation of gelation [39,40,41]. Secondly, sugars can conjugate with proteins through the Maillard reaction, which is caused by a carbonyl-amine reaction between reducing sugars and proteins during heat treatment [24]. Furthermore, protein aggregation could be influenced by steric effects because of the highly-branched chain of SSPS, which might lead to the degradation of the characteristics of tofu gelation [8]. When the concentration of SSPS increased from 8% to 12%, the gel strength decreased significantly. At this point, SSPS had a greater effect on the destruction of SPI gels to some extent.

#### 3.4.3. Change in the Microstructure of SPI Gels

**Figure 6:**
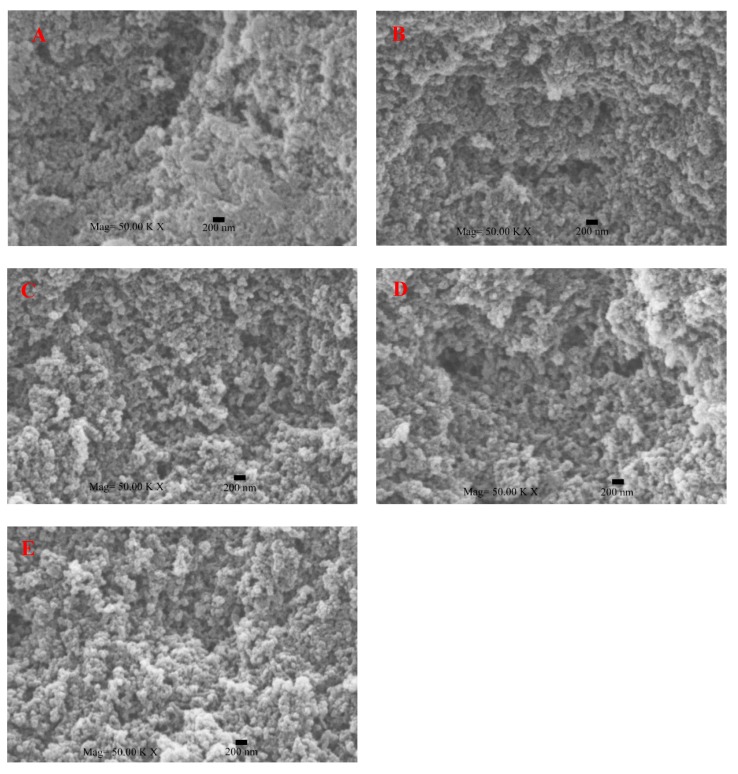
Scanning electron microscope (SEM) of SSPS–SPI gels with different SSPS concentrations (SPI, soybean protein isolate; SSPS, soybean soluble polysaccharide; (**A**) 0%; (**B**) 4%; (**C**) 8%; (**D**) 12%; (**E**) 16%).

As shown in Figure 6, as for the control, there was an uneven structure with many small irregular pores occupying the space of the gel matrix. It was clear that there were significant differences between the image of 16% SSPS-added gel and the control. Their microstructures were much laxer and more poriferous than that of the control. Overall, the pore sizes and particle size were bigger when the SSPS concentration was higher. Wang et al. [37] reported that exposure of more hydrophobic groups contributes to the formation of a denser gel network structure. The size and shape of protein molecules might affect the microstructure of protein [42]. Furthermore, the microstructure was closely related to gelation and aggregation [43]. Gels with bigger aggregates and bigger holes in the gel microstructure usually have less gel strength. In other words, SSPS retard the formation of SPI gels and result in SPI gels with looser microstructure and lower WHC and gel strength. This was due to the lower gelling rate aggravating the phase separation phenomenon by adding the negative charge of polysaccharides, resulting in an increase of microstructure porosity and weaker gels [7].

### 3.5. Effect of SSPS on the Formation of GDL-Induced SPI Gels

**Figure 7:**
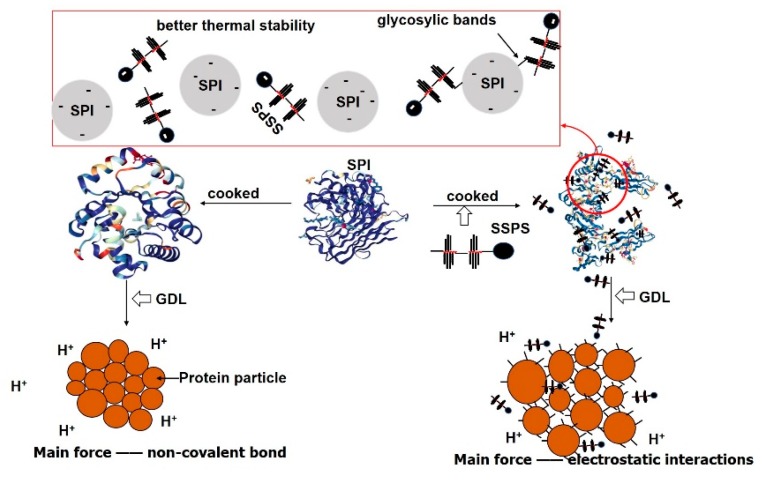
The proposed mechanism of the formation of GDL-induced SPI gel when SSPS was added (SPI, soybean protein isolate; SSPS, soybean soluble polysaccharide).

The possible mechanism for the effect of SSPS on the GDL-induced SPI gelation is shown in Figure 7. SSPS and SPI might conjugate via the Maillard reaction according to the results of grafting degree, color change, and infrared analyses. As a result, the structure of cooked SPI suspension was altered by adding SSPS. The disulfide bond is an important intermolecular force for the stiffness and solidness of SPI gels, and specifically for the high density of their protein networks [44]. In addition, hydrophobic groups and hydrogen bonds are very important for the formation of soybean protein gels [45]. The addition of SSPS could lead to weaker electrostatic interactions [27] and stronger non-covalent interactions (such as hydrogen bonding) in cooked SPI; at the same time the content of free sulfhydryl groups increased. SSPS can improve the thermal stability of the SPI by molecular repulsive forces to make the solution more stable and prevent the heat denaturation of the protein. The increase in content of the β-sheet increased, and the reduction of contents of α-helix and random coil were found in the cooked SPI. After adding GDL, electrostatic interactions were enhanced, non-covalent bonds were weakened, and the content of the free sulfhydryl group was still higher than that of the control group. However, the change trends of the surface hydrophobicity and secondary structure of SPI did not change. Finally, as the SSPS was added, the main force of the SPI gel network changed from non-covalent bonds to electrostatic interactions, and the gel strength showed an obvious downward trend. Furthermore, protein aggregation could be influenced by steric effects because of the highly-branched chain of SSPS, which might lead to the degradation of the characteristics of tofu gelation [8]. As a result, there was a loose network in GDL-induced SPI gels when SSPS was added.

## 4. Conclusions

These results pointed out that the addition of SSPS had a profound influence on the gelling properties of SPI gels by decreasing their WHC and gel strength and changing their microstructure. The changes in functional features mentioned above could be due to the changes in structural and functional properties induced by SSPS. In summary, SSPS increased the β-sheet content and decreased the contents of random coil and α-helix in both cooked SPI and SPI gel, and increased hydrophobic interactions of SPI gels. SSPS might conjugate with SPI via the Maillard action. Furthermore, the addition of SSPS reduced the content of the sulfhydryl group of cooked SPI suspension and changed the main intermolecular forces of the gel network from non-covalent bonds to electrostatic interactions. There were fewer gel aggregates formed and a structure of looser and increased porosity in SPI gels. However, the effect of glycosylation products between SSPS and proteins on the formation of SPI gels needs further study.

## Figures and Tables

**Table 1:** Percentage of secondary structure distribution of cooked SPI suspension and SPI gels (%).

SSPS Concentration (%)	Cooked SPI Suspension				SPI Gels			
	β-Sheet	Random Coil	α-Helix	β-Turns	β-Sheet	Random Coil	α-Helix	β-Turns
0	22.99	28.24	23.17	25.6	24.63	2.79	45.14	27.44
4	27.86	21.33	10.25	40.56	37.08	0.06	36.71	26.15
8	35.66	20.83	14.06	29.45	38.72	0	28.79	32.49
12	36.33	14.7	13.62	35.35	42.55	0.01	29	28.44
16	42.14	7.43	5.67	44.76	33.07	0	39.01	27.92

**Table 2:** Solubility of cooked SPI suspension and SPI gels in different buffers (%) ^a, b^.

SSPS Concentration (%)	Cooked SPI Suspension					SPI Gels				
	S1	S2	S3	S2–S1	S3–S2	S1	S2	S3	S2–S1	S3–S2
0	28.6 ± 0.3c	68.9 ± 0.5a	80.2 ± 0.8d	40.2	11.3	16.1 ± 0.2d	37.8 ± 1.5b	80.5 ± 1.8c	21.7	42.7
4	23.7 ± 1.5d	63.2 ± 0.7b	88.0 ± 1.2b	39.5	24.8	19.2 ± 1.3c	81.2 ± 2.9a	82.8 ± 0.5c	62.1	1.6
8	24.6 ± 1.4d	63.2 ± 0.4b	93.5 ± 1.5a	38.5	30.4	28.7 ± 0.2a	83.9 ± 1.8a	82.7 ± 0.3c	55.2	1.2
12	30.5 ± 0.2b	61.8 ± 0.1c	84.3 ± 1.2c	31.3	22.5	23.2 ± 0.4b	85.4 ± 1.2ab	86.5 ± 1.9a	63.3	1.1
16	36.7 ± 1.3a	62.7 ± 0.1bc	81.1 ± 0.5d	25.9	18.4	23.3 ± 0.8b	86.1 ± 0.5a	87.5 ± 1.5a	64.2	1.4

^a^ The data are expressed as mean ± standard deviations (n = 3). Results with different letters in the same column are significantly different (*p* < 0.05). ^b^ Abbreviations: S1, deionized water at pH 8.0 (adjusted with NaOH); S2, Tris–Glycine buffer (0.086 mol/L Tris, 0.09 mol/L glycine, and 4 mmol/L Na_2_EDTA, pH 8.0); S3, S2 containing 0.5% sodium dodecyl sulphate and 6 mol/L urea; SPI, soybean protein isolate.

**Table 3:** DG and L*, a*, and b* of SPI gels with different SSPS concentrations ^a, b^.

SSPS Concentration (%)		Color of SPI Gels		
	DG of SPI Gels	L*	a*	b*
0	0	41.5 ± 0.3c	3.5 ± 0.5c	6.5 ± 0.2c
4	2.4 ± 0.2d	41.6 ± 0.3c	3.3 ± 0.5c	7.4 ± 0.3b
8	3.8 ± 0.02c	43.3 ± 0.7b	4.9 ± 0.5b	7.6 ± 0.2b
12	4.5 ± 0.1b	44.4 ± 0.5a	7.3 ± 0.7a	9.1 ± 0.3a
16	8.5 ± 0.2a	44.4 ± 0.5a	7.4 ± 0.6a	9.2 ± 0.3a

^a^ The data are expressed as mean ± standard deviations (*n* = 3). Results having different letters in one column are significantly different (*p* < 0.05). ^b^ Abbreviations: DG, degree of grafting; SPI, soybean protein isolate.

## Supplementary Materials

The following are available online at https://www.mdpi.com/2073-4360/11/12/1997/s1, Figure S1: FTIR spectra of CSPI suspension (A) and SPIG (B) as the SSPS concentration increased (SPI, soybean protein isolate; SSPS, soybean soluble polysaccharide; CSPI, cooked SPI; SPIG, SPI gels).
