# Supplementary material for: Effect of Soybean Soluble Polysaccharide on the Formation of Glucono-δ-Lactone-Induced Soybean Protein Isolate Gel
Source: Polymers (Basel). 2019 Dec 3;11(12):1997. doi: 10.3390/polym11121997 (PMC6960500; doi:10.3390/polym11121997)
Supplement: Supplementary file 1 [file polymers-11-01997-s001.pdf]

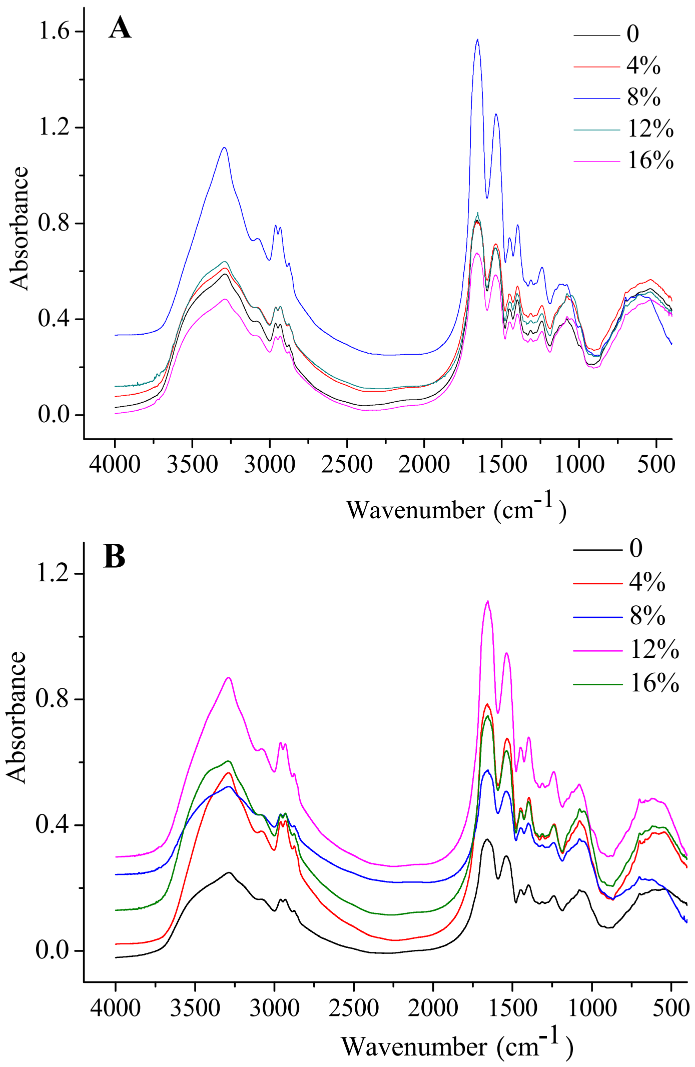

Figure S1: FTIR spectra of CSPI suspension (A) and SPIG (B) as the SSPS concentration increased (SPI, soybean protein isolate; SSPS, soybean soluble polysaccharide; CSPI, cooked SPI; SPIG, SPI gel).
